# Factors associated with preconception care behaviors and behavioral stages among emerging adults: a cross-sectional study

**DOI:** 10.3389/fpubh.2026.1842698

**Published:** 2026-08-13

**Authors:** Inhae Cho, Hyunjeong Shin, Songi Jeon

**Affiliations:** 1 College of Nursing, Inje University, Busan, Republic of Korea; 2 College of Nursing, Korea University, Seoul, Republic of Korea; 3 Department of Nursing, Catholic Kwandong University, Gangneung, Republic of Korea

**Keywords:** health behavior, patient acceptance of health care, preconception care, reproductive health, stages of change

## Abstract

**Background:**

It remains a challenge to promote reproductive healthcare behaviors—including preconception care—among emerging adults. To effectively influence behavioral changes among emerging adults, it is necessary to understand their behavior according to their psychological steps.

**Objective:**

This study aimed to describe the distribution of preconception care behavioral stages among emerging adults, using the framework of the precaution adoption process model (PAPM), and to examine factors associated with each stage.

**Methods:**

This cross-sectional study used an online convenience sample of 929 participants aged 18–25 years in South Korea between March and October 2020. Behavioral stages of preconception care were assessed using the PAPM, and participants were classified into three groups (unaware, pre-adoption, and adoption of preconception care behaviors). Separate binary logistic regression models were used to compare each group with the adoption group as a reference.

**Results:**

Compared with the adoption group, the unaware group was more likely to be male, to be sexually inexperienced, to use contraception inconsistently, to have no intention of future childbearing, and to report higher self-perceived knowledge of pregnancy-related issues. The pre-adoption group was more likely to be sexually inexperienced and to report lower perceived health status and lower levels of reproductive health-promoting behaviors.

**Conclusions:**

This study described the behavioral stages of preconception care using the PAPM framework and identified characteristics associated with each stage. The findings may inform the development of future communication strategies; however, further research is required to evaluate their effectiveness.

## 1 Introduction

Beyond prenatal care, attention to preconception health among both women and men is increasingly recognized as a critical component of reproductive health. Preconception care refers to biomedical, behavioral, and social interventions implemented prior to conception to optimize health outcomes for parents and offspring (1). Maternal health status and behaviors during the preconception period—such as nutrition, physical activity, and substance use—have been shown to affect fertility, embryonic development, and long-term child health (2–4). Consequently, promoting healthy behaviors before pregnancy can improve reproductive outcomes and reduce the risk of adverse maternal and neonatal conditions.

Despite its importance, many individuals remain unaware of how their lifestyle choices influence reproductive and pregnancy outcomes (5, 6). This lack of awareness is particularly evident among emerging adults, who often have limited access to reproductive health education and services (7, 8). This is also the case in South Korea, where sexual and reproductive health education tailored to university-aged adults remains limited (9, 10). In addition, Korea is undergoing a shift toward later marriage and childbearing, with the mean maternal age at first birth (33.1 years in 2024), the highest among OECD countries (11, 12). Rather than placing emerging adults at immediate obstetric risk, this shift lengthens the interval between sexual maturity and eventual childbearing, making emerging adulthood an extended preconception window in which the health behaviors established at this stage may shape reproductive outcomes years later. This is especially relevant given that nearly half of all pregnancies worldwide are unintended (13), with a disproportionate share occurring among individuals aged 18–29 (14).

Emerging adulthood, a developmental stage spanning approximately ages 18–25 that is marked by identity exploration, instability, self-focus, and a sense of being in-between adolescence and adulthood (15), is a crucial period for establishing long-term health behaviors (16, 17). We focused on the 18–25 range because, in South Korea, it is a period still distant from employment and childbearing yet one in which sexual debut typically occurs (18). Reproductive health risk exposure, therefore, begins well before pregnancy is anticipated, making emerging adulthood an opportune period to establish preconception care behaviors.

The Precaution Adoption Process Model (PAPM) is a psychological stage model that describes the sequence of mental states individuals pass through before adopting a new health behavior (19). Unlike models that assume linear behavior change, the PAPM identifies early stages such as unawareness and passive decision-making, making it particularly suitable for emerging adults who may lack foundational knowledge about reproductive health. By classifying individuals according to their behavioral stage, the PAPM has been proposed to inform interventions that address stage-specific needs and barriers. However, stage-based models have rarely been applied to preconception care. Although PAPM-based, stage-tailored programs have been shown to improve reproductive health-promoting behaviors in Korean young women (20), it remains unknown how Korean emerging adults are distributed across PAPM stages for preconception care, and which sociodemographic and behavioral factors distinguish those who are unaware of or undecided about it from those who have begun to adopt it. Characterizing this distribution and these factors may provide useful information for future studies investigating stage-specific approaches.

Therefore, this study aimed to apply the PAPM to classify emerging adults aged 18–25 in South Korea by their behavioral stage for preconception care and to examine the sociodemographic and behavioral factors associated with each stage. In particular, we sought to characterize the early-stage groups—those unaware of or not yet engaged in preconception care—to inform stage-matched strategies for promoting reproductive health behaviors in this population.

## 2 Methods

### 2.1 Study design and participants

This study adopted a cross-sectional design to identify the factors and stages related to preconception care among emerging adults in South Korea. Data were collected using convenience sampling through an online survey administered between March 27 and October 19, 2020. We recruited participants through an Instagram promotion post and the notice board of an online community associated with a university based in Seoul. The inclusion criteria were as follows: (1) emerging adults aged 18–25 years, and (2) no language barriers. The exclusion criteria were as follows: (1) currently pregnant or previous childbirth experience, and (2) foreign nationality or difficulty communicating in Korean. Participants who were pregnant or had given birth were excluded because this study focused on the period before the first pregnancy. In people who are already pregnant or have a child, the PAPM stage may reflect their pregnancy experience rather than their readiness to begin preconception care. A total of 1,124 individuals completed the online survey, and 929 participants were included in the final analysis after excluding surveys with incomplete responses.

### 2.2 Measures

#### 2.2.1 PAPM stages in preconception care

To categorize the PAPM stages in preconception care, we included the following questions in the survey (19, 21):

- Have you heard of preconception care?
- Do you engage in relevant behaviors to improve your health or reproductive health for preconception care?
- What best describes your thoughts on preconception health and care?

After obtaining the responses to these questions, we classified the PAPM stages in preconception care according to the answers. The original PAPM consists of seven stages (“maintenance” stage), but this study focused on individuals' decisions to adopt and engage in preventive behaviors. Therefore, we considered only the first six stages of the PAPM—from “unaware” to “acting.” For analytical purposes, the stages were grouped based on behavioral readiness. The PAPM stages were further categorized into three groups: unaware (Stage 1), pre-adoption (Stages 2 and 3), and adoption (Stages 5 and 6). Stages 2 (unengaged) and 3 (undecided) were combined into a “pre-adoption” group because both represent individuals who are aware of preconception care but have not yet decided to adopt it—neither committed to action nor acting; Stage 3 alone (*n* = 15) was also too small for separate analysis. Stages 5 (decided to act) and 6 (acting) were combined into an “adoption” group because both reflect a positive behavioral commitment toward preconception care, in contrast to unawareness (Stage 1) and the unengaged/undecided states (Stages 2–3). This group was overwhelmingly composed of participants already acting (Stage 6, *n* = 609; 95.9%), with only 26 (4.1%) at Stage 5. Stage 4 (decided not to act) was excluded as only two participants were so classified. The full staging questions and classification rules are presented in Supplementary Table S1.

#### 2.2.2 Health behavior factors

Health-related behaviors assessed in this study included perceived health status, BMI, dietary habits, physical activity, smoking, alcohol use, sleep duration, reproductive health-promoting behaviors, and sexual behavior. Most items were adapted from the Korea Youth Risk Behavior Web-Based Survey (22). Physical activity was evaluated based on World Health Organization (WHO) guidelines (23), and heavy alcohol use was defined as consuming more than four drinks for men and three for women on any day (24).

Dietary habits were assessed using a 41-item checklist developed by the Korean Ministry of Food and Drug Safety (25), covering high-fat/calorie intake, binge eating, impulsive eating, meal skipping, and sodium intake. Responses in this scale were rated on a five-point Likert scale, with higher scores indicating more risky eating behaviors (Cronbach's α: 0.51–0.90).

Reproductive health-promoting behaviors were measured using a gender-specific scale (26) comprising 18 items for females and 16 for males, each rated on a four-point Likert scale. Because the two versions differ in length, scores were computed as the mean item score (range 1–4) rather than as summed totals, so that values are directly comparable across genders (higher scores indicate healthier behaviors; Cronbach's α: 0.91–0.99).

#### 2.2.3 Psychosocial factors

Four psychosocial factors were assessed: future family planning, self-perceived knowledge, objective knowledge, and awareness of preconception care. Future family planning was evaluated with a single question on childbearing intention. Self-perceived knowledge was a subjective self-appraisal of how knowledgeable participants felt about pregnancy-related issues, measured with two items adapted from Delgado (27) on a four-point scale (not at all to extremely aware; mean range 1–4); higher scores indicate greater perceived knowledge—how knowledgeable participants feel—rather than objective knowledge or perceived risk (Cronbach's α = 0.73). Objective knowledge was measured with 17 multiple-choice items scored as the number of correct answers (range 0–17), also adapted from Delgado (27); higher scores indicate greater actual knowledge. Consistent with the original instrument, the self-perceived knowledge items were kept separate from the objective knowledge score, as the two represent distinct constructs. Awareness of men's and women's preconception care was measured with a statement regarding the impact of preconception health on pregnancy outcomes, with responses categorized as “yes,” “no,” or “I do not know.”

### 2.3 Data analysis

We analyzed the collected data using STATA 17.0 (StataCorp LLC, College Station, TX, USA). Descriptive statistics were reported using frequencies, percentages (%), means, and standard deviations (SD) for all variables. We used a chi-square test and analysis of variance (ANOVA) to examine the differences between the factors according to the PAPM stages. Finally, we used Scheffé's test for *post-hoc* analysis. Multicollinearity among the predictors was assessed using variance inflation factors; all VIFs were below 2.1 (mean 1.44), indicating no problematic collinearity.

Using the adoption group as the reference category, binary logistic regression was used to estimate each contrast—unaware versus adoption and pre-adoption versus adoption—in a separate model. Participants in Stage 4 (*n* = 2) were excluded because of the very small number of observations. For each contrast, unadjusted associations were first estimated for every candidate variable. Variables significant in the bivariate analyses (*p* < 0.05), together with theoretically relevant variables, were then entered into multivariable models. For the unaware contrast, Model 1 included all participants (using ever having had sexual intercourse), and Model 2 was restricted to sexually experienced participants so that contraceptive use could be entered. Adjusted odds ratios (aORs) and 95% CIs were estimated, with significance set at *p* < 0.05 (two-tailed).

Sample adequacy was evaluated using the events-per-variable (EPV) criterion of at least 10 events per estimated parameter (28), applied to the smaller outcome group in each model. The unaware-vs.-adoption model with all participants (Model 1; 11 parameters) and the pre-adoption-vs.-adoption model (3 parameters) each exceeded this threshold; the sexually experienced subgroup model (Model 2; 13 parameters) had the lowest EPV, and its estimates are interpreted with caution.

### 2.4 Ethical approval

The study protocol was approved by the Institutional Review Board of our university (No. KUIRB-2019-0337-04). All potential participants were provided with adequate information about the study's objectives and procedures, confidentiality, benefits, and risks of participation. We also explained the anonymous data collection process and right to withdraw, including the right to skip any question or stop at any time if psychologically uncomfortable. After providing this information, we asked each participant to voluntarily sign an online consent form. Participants were only allowed to complete the questionnaire after signing the consent forms.

## 3 Results

### 3.1 Demographic characteristics

Regarding the PAPM stages of preconception health care, 219 (23.6%), 58 (6.2%), 15 (1.6%), 2 (0.2%), 26 (2.8%), and 609 (65.6%) participants were in Stages 1, 2, 3, 4, 5, and 6, respectively.

To examine the decision-making processes and behaviors related to preconception care, the PAPM stages were analyzed as three groups (unaware, pre-adoption, and adoption). Participants in Stage 4 (deciding not to act) were excluded from regression analyses due to the extremely small sample size (*n* = 2), which could compromise statistical stability.

We analyzed the responses of 929 participants with a mean age of 21.67 years, 83.9% of whom were female (*n* = 779). Gender was the only characteristic that differed significantly across the three PAPM groups (*p* = 0.012); no other demographic variable differed. Table 1 presents a detailed list of the participants' demographic characteristics.

**Table 1:** General characteristics of the study populations according to PAPM stages.

Characteristic	Total (*n* = 929)	Unaware group (*n* = 219)	Pre-adoption group (*n* = 73)	Adoption group (*n* = 635)	X^2^ or F (*p*)
	Mean ±SD or *n* (%)				
Age	21.67 ± 2.06	21.46 ± 2.19	21.44 ± 2.09	21.77 ± 2.01	2.38 (0.093)
Gender					8.86 (0.012)
Female	779 (83.9)	170 (77.6)	60 (82.2)	547 (86.1)	
Male	150 (16.1)	49 (22.4)	13 (17.8)	88(13.9)	
Marital status					0.02 (0.991)
Married	12 (1.3)	3 (1.4)	1 (1.4)	8 (1.3)	
Unmarried	917 (98.7)	216 (98.6)	72 (98.6)	627 (98.7)	
Economic level					4.01 (0.405)
Low	217 (23.4)	57 (26.0)	21 (28.8)	139 (21.9)	
Middle	669 (72.0)	153 (69.9)	47 (64.4)	467 (73.5)	
High	43 (4.6)	9 (4.1)	5 (6.9)	29 (4.6)	
Education					7.15 (0.128)
High school graduate or lower	584 (62.8)	151 (68.9)	49 (67.1)	383 (60.3)	
College graduate	336 (36.2)	65 (29.7)	23 (31.5)	247 (38.9)	
Graduate or higher	9 (1.0)	3 (1.4)	1 (1.4)	5 (0.8)	
Employment status					1.52 (0.958)
High school student	29 (3.1)	8 (3.7)	3 (4.1)	18 (2.8)	
Undergraduate or graduate student	595 (64.1)	144 (65.8)	48 (65.8)	402 (63.3)	
Employed	160 (17.2)	35 (16.0)	12 (16.4)	112 (17.6)	
Jobseeker	145 (15.6)	32 (14.6)	10 (13.7)	103(16.2)	

The total (*n* = 929) includes two participants in Stage 4 (decided not to act), who were excluded from the group comparisons (analytic *n* = 927).

### 3.2 Health behaviors and psychosocial factors

Among health behaviors, perceived health status, reproductive health-promoting behaviors, and sexual behaviors showed significant differences across the three PAPM groups (Table 2). The pre-adoption group had the lowest perceived health score (2.90 ± 1.27), and all groups exhibited generally low levels in reproductive health-promoting behaviors (3.40 ± 0.40).

**Table 2:** Health behaviors and psychosocial factors according to PAPM stages (*n* = 927).

Factors	Total (*n* = 927)	Unaware group (*n* = 219)	Pre-adoption group (*n* = 73)	Adoption group (*n* = 635)	X^2^ or F (*p*)
	Mean ±SD or *n* (%)				
Health behavior factors					
Perceived health status^a^	3.32 ± 1.14	3.30 ± 1.22	2.90 ± 1.27	3.38 ± 1.09	5.77 (0.003)
BMI					
Underweight (<18.5 kg/m^2^)	131 (14.1)	29 (13.2)	12 (16.4)	90 (14.2)	0.46 (0.793)
Overweight/obesity (>25 kg/m^2^)	147 (15.8)	38 (17.4)	17 (23.3)	91 (14.3)	4.51 (0.105)
Dietary habits					
High-fat/high-calorie	3.11 ± 0.62	3.10 ± 0.65	3.18 ± 0.61	3.10 ± 0.62	0.59 (0.559)
Overeating/binge eating	2.91 ± 0.79	2.90 ± 0.78	3.08 ± 0.79	2.90 ± 0.79	1.67 (0.189)
Impulsive eating behavior	2.80 ± 0.91	2.78 ± 0.92	3.02 ± 0.90	2.78 ± 0.91	2.25 (0.106)
Skipping meals	3.15 ± 0.79	3.10 ± 0.82	3.20 ± 0.83	3.17 ± 0.78	0.62 (0.537)
Sodium intake	2.77 ± 0.59	2.75 ± 0.57	2.84 ± 0.59	2.77 ± 0.60	0.55 (0.577)
Sedentary time (hours/day)	7.10 ± 3.08	7.28 ± 3.37	6.85 ± 2.99	7.06 ± 3.00	0.64 (0.526)
Insufficient physical activity^b^	754 (81.2)	177 (80.8)	63 (86.3)	512 (80.6)	1.39 (0.499)
Current smoking (>1 cigarette/day)	63 (6.8)	19 (8.7)	3 (4.1)	41 (6.5)	2.17 (0.338)
Heavy alcohol use during the last month^c^	431 (46.4)	90 (41.1)	32 (43.8)	309 (48.7)	3.97 (0.137)
Sleep duration (hours/day)	7.79 ± 1.27	7.77 ± 1.32	7.85 ± 1.21	7.79 ± 1.26	0.12 (0.890)
Reproductive health-promoting behavior	3.40 ± 0.40	3.36 ± 0.42	3.30 ± 0.41	3.43 ± 0.39	4.61 (0.010)
Sexual behaviors					
Ever had sexual intercourse (*n* = 878)					
Yes	584 (66.6)	112 (56.3)	34 (48.6)	437 (72.0)	27.67 (<0.001)
No	294 (33.5)	87 (43.7)	36 (51.4)	170 (28.0)	
Contraception (*n* = 584)					
Always	281 (48.1)	47 (42.0)	15 (44.1)	218 (49.9)	25.31 (<0.001)
Mostly	211 (36.1)	39 (34.8)	16 (47.1)	156 (35.7)	
Sometimes	72 (12.3)	14 (12.5)	2 (5.9)	56 (12.8)	
Not at all	20 (3.4)	12 (10.7)	1 (2.9)	5 (1.6)	
Psychosocial factors					
Future family planning					34.91 (<0.001)
I will surely have children	161 (17.3)	40 (18.3)	13 (17.8)	108 (17.0)	
I think it is good to have children	253 (27.2)	50 (22.8)	11 (15.0)	192 (30.2)	
I do not care if I have children in the future	316 (34.0)	62 (28.3)	27 (37.0)	227 (35.8)	
I will never have children	102 (11.0)	42 (19.2)	10 (13.7)	48 (7.6)	
I do not know	97 (10.4)	25 (11.4)	12 (16.4)	60 (9.5)	
Self-perceived knowledge	2.56 ± 0.79	2.97 ± 0.76	2.57 ± 0.76	2.41 ± 0.76	42.84 (<0.001)
Objective knowledge	11.40 ± 2.15	11.03 ± 2.11	11.51 ± 2.32	11.52 ± 2.14	4.32 (0.014)
Awareness of preconception care					
If men take care of their health before conception, they are more likely to have a healthy pregnancy and child (‘Yes')	864 (93.0)	190 (86.8)	70 (95.9)	602 (94.8)	17.19 (<0.001)
If women take care of their health before conception, they are more likely to have a healthy pregnancy and child (‘Yes')	895 (96.3)	204 (93.2)	71 (97.3)	618 (97.3)	8.22 (0.016)

SD, standard deviation. Ever had sexual intercourse, *n* = 878; contraception, *n* = 584 (asked only of sexually experienced participants). ^a^Range from 1 (very unhealthy) to 5 (very healthy). ^b^Percentage of participants who did not meet the World Health Organization recommendations (≥150 min of moderate-intensity or ≥75 min of vigorous-intensity physical activity per week, or an equivalent combination). ^c^ Percentage of participants who consumed more than four drinks on any day for men or more than three drinks for women in the past month.

Nutritional status varied by gender, with 15.7% of females and 6.0% of males underweight, while 15.0% of females and 20.0% of males were overweight or obese. Among dietary behaviors, skipping meals had the highest risk score (3.15 ± 0.79). In addition, 81.2% of participants reported insufficient physical activity. The mean sedentary time was 7.10 h per day, and the average sleep duration was 7.79 h per day. Nearly half (46.4%) reported heavy alcohol use during the past month, whereas only 6.8% smoked daily.

Of the 878 participants who responded to sexual behavior items, 584 had sexual experience, 48.1% reported “always,” and 36.1% reported “mostly” using contraception. The adoption group had the highest sexual experience rate (72%) and consistent contraceptive use (49.9%).

Regarding future family planning, 17.3% of participants intended to have children in the future, while 11.0% reported no intention of childbearing. The mean knowledge score was 11.4 out of 17 (67.1% correct). Most participants acknowledged the benefits of preconception care for women (96.3%) and men (93.0%).

### 3.3 Bivariate analysis on factors associated with the PAPM stages in preconception care

Compared with the adoption group (Table 3), the unaware group had higher odds of being male (OR 1.79, 95% CI 1.21–2.64), having no sexual experience (OR 2.00, 95% CI 1.43–2.78), engaging in unprotected sex (OR 7.95, 95% CI 2.97–21.27), having no desire for children (OR 2.36, 95% CI 1.36–4.10), and reporting higher self-perceived knowledge of pregnancy-related issues (OR 2.60, 95% CI 2.08–3.25), as well as lacking awareness of preconception care for both men (OR 2.78, 95% CI 1.65–4.71) and women (OR 2.67, 95% CI 1.31–5.45). They also had lower odds of engaging in reproductive health-promoting behavior (OR 0.66, 95% CI 0.45–0.96) and objective knowledge scores (OR 0.90, 95% CI 0.84–0.97).

**Table 3:** Unadjusted binary logistic regression of factors associated with preconception-care stages, with the adoption group as the reference category (*n* = 927).

Factors	Categories	UG (vs. AG)	PG (vs. AG)
		OR (95% CI)	
Sex			
Male (Ref. = female)		1.79 (1.21–2.64)	1.35 (0.71–2.56)
Perceived health status^a^		0.94 (0.82–1.08)	0.71 (0.58–0.87)
Ever had sexual intercourse (*n* = 878)			
No (Ref. = Yes)		2.00 (1.43–2.78)	2.72 (1.65–4.49)
Contraception (*n* = 584)			
Always (Ref.)			
Mostly		1.16 (0.72–1.86)	1.49 (0.72–3.10)
Sometimes		1.16 (0.60–2.25)	0.52 (0.12–2.37)
Not at all		7.95 (2.97–21.27)	2.08 (0.24–18.00)
Future family planning			
I will surely have children (Ref.)			
I think it is good to have children		0.70 (0.44–1.13)	0.48 (0.21–1.10)
I do not care if I have children in the future		0.74 (0.47–1.17)	0.99 (0.49–1.99)
I will never have children		2.36 (1.36–4.10)	1.73 (0.71–4.22)
I do not know		1.13 (0.62–2.03)	1.66 (0.71–3.87)
Reproductive health-promoting behavior		0.66 (0.45–0.96)	0.47 (0.26–0.86)
Self-perceived knowledge		2.60 (2.08–3.25)	1.31 (0.95–1.80)
Objective knowledge		0.90 (0.84–0.97)	1.00 (0.89–1.12)
Awareness of preconception care^b^			
Men's preconception care			
No (Ref. = Yes)		2.78 (1.65–4.71)	0.78 (0.23–2.62)
Women's preconception care			
No (Ref. = Yes)		2.67 (1.31–5.45)	1.02 (0.23–4.52)

AG, adoption group (Stages 5–6); PG, pre-adoption group (Stages 2–3); UG, unaware group (Stage 1); OR, odds ratio; CI, confidence interval. Unaware vs. adoption, *n* = 854; pre-adoption vs. adoption, *n* = 708. ^a^Range from 1 (very unhealthy) to 5 (very healthy). ^b^Participants answered: “If men (or women) take care of their health before conception, they are more likely to have a healthy pregnancy and child.”

The pre-adoption group was more likely than the adoption group to have no sexual experience (OR 2.72, 95% CI 1.65–4.49), perceive poorer health status (OR 0.71, 95% CI 0.58–0.87), and report lower scores in reproductive health-promoting behaviors (OR 0.47, 95% CI 0.26–0.86).

### 3.4 Multivariable analysis on factors associated with the PAPM stages in preconception care

We conducted multivariable logistic regression to identify factors associated with preconception-care behaviors among emerging adults (Table 4). To compare the characteristics of the unaware and adopted groups, we constructed two models based on sexual experience: Model 1 included all participants, and Model 2 included only those who had engaged in sexual intercourse.

**Table 4:** Adjusted (multivariable) binary logistic regression of factors associated with preconception-care stages, with the adoption group as the reference category (*n* = 927).

Factors	Categories	UG (vs. AG)		PG (vs. AG)
		Model 1	Model 2	
		aOR (95% CI)		
Sex				
Male (Ref. = female)		1.79 (1.13–2.82)	1.72 (0.98-3.04)	–
Perceived health status^a^		-	-	0.71 (0.58–0.88)
Ever had sexual intercourse				
No (Ref. = Yes)		1.74 (1.19–2.54)	-	3.16 (1.87–5.34)
Contraception				
Always (Ref.)		-	-	-
Mostly		-	0.99 (0.59–1.69)	-
Sometimes		-	1.03 (0.48–2.21)	-
Not at all		-	6.10 (2.01–18.45)	-
Future family planning				
I will surely have children (Ref.)				-
I think it is good to have children		0.66 (0.39–1.15)	0.55 (0.28–1.08)	-
I do not care if I have children in the future		0.69 (0.41–1.17)	0.60 (0.31–1.15)	-
I will never have children		2.32 (1.23–4.38)	2.63 (1.16–5.98)	-
I do not know		1.01 (0.51–1.97)	0.80 (0.31–2.06)	-
Reproductive health-promoting behavior		0.77 (0.50–1.22)	0.70 (0.38–1.27)	0.36 (0.19–0.70)
Self-perceived knowledge		2.48 (1.93–3.18)	2.47 (1.77–3.46)	-
Objective knowledge		0.98 (0.90–1.06)	0.97 (0.87–1.08)	-
Awareness of preconception care^b^				
Men's preconception care				
No (Ref. = Yes)		1.86 (0.86–4.02)	1.16 (0.38–3.56)	-
Women's preconception care				
No (Ref. = Yes)		1.16 (0.40–3.33)	1.28 (0.32–5.17)	-

AG, adoption group (Stages 5–6); PG, pre-adoption group (Stages 2–3); UG, unaware group (Stage 1); aOR, adjusted odds ratio; CI, confidence interval. For the unaware contrast, Model 1 included all participants (*n* = 806) and Model 2 only those sexually experienced (*n* = 549); the pre-adoption contrast was a single model (*n* = 677). ^a^Range from 1 (very unhealthy) to 5 (very healthy). ^b^Awareness of preconception care was assessed using a single-item question. −Variable not included in the model.

Perceived health status was measured on a 5-point scale (1 = very unhealthy to 5 = very healthy); higher scores indicate better perceived health.In Model 1, the unaware group was more likely to be male (aOR 1.79, 95% CI 1.13–2.82), to have no experience of sexual intercourse (aOR 1.74, 95% CI 1.19–2.54), to lack the desire to have children (aOR 2.32, 95% CI 1.23–4.38), and to report higher self-perceived knowledge of pregnancy-related issues (aOR 2.48, 95% CI 1.93–3.18). In Model 2, the unaware group was characterized by never using contraception (aOR 6.10, 95% CI 2.01–18.45), no desire for future children (aOR 2.63, 95% CI 1.16–5.98), and higher self-perceived knowledge (aOR 2.47, 95% CI 1.77–3.46).

The pre-adoption group was more likely to have never engaged in sexual intercourse (aOR 3.16, 95% CI 1.87–5.34), to report lower perceived health status (aOR 0.71, 95% CI 0.58–0.88), and to report lower reproductive health-promoting behavior scores (aOR 0.36, 95% CI 0.19–0.70).

## 4 Discussion

We explored factors associated with preconception care behaviors among emerging adults aged 18–25, using the Precaution Adoption Process Model (PAPM) to categorize participants into three behavioral stages: unaware, pre-adoption, and adoption. To our knowledge, this is the first study to apply the PAPM framework in examining factors associated with preconception health behavior among emerging adults. Our findings provide descriptive evidence on the distribution of stages and their associated characteristics.

Our findings revealed that the unaware group was more likely to be male, to have no sexual experience, to never use contraception, to lack intention for future childbearing, and to report higher self-perceived knowledge of pregnancy-related issues despite no higher objective knowledge and lower engagement in reproductive health-promoting behaviors. The pre-adoption group was characterized by a lack of sexual experience, lower perceived health status, and relatively low reproductive health-promoting behavior scores.

Sexual experience differed across behavioral stages. Participants without sexual experience were more likely to belong to unaware or pre-adoption groups. One possible explanation is that these individuals may have had fewer opportunities to encounter sexual and reproductive health information. However, because this study did not assess exposure to sexual health education, counseling, or health services, this interpretation remains speculative and should be examined in future research. Nevertheless, preconception health should be promoted as a fundamental issue for all emerging adults, regardless of sexual activity status (1, 29, 30). The adoption group's higher rate of sexual experience and more consistent contraceptive use may indicate that preconception care—and current preconception health messaging—is perceived as relevant primarily after sexual debut rather than before it. Likewise, participants who never used contraception were more likely to be in the unaware group. Because contraception and preconception care together span the reproductive life course and are most effective when addressed in an integrated way (31), non-use of contraception may reflect not only limited awareness but also fertility intentions, access, or relationship circumstances that our data cannot disentangle. This underscores the need for both broad awareness-raising and integrated reproductive health support that links contraception and preconception care.

A notable finding was that the unaware group reported higher self-perceived knowledge of pregnancy-related issues, even though their objective knowledge was no higher and they engaged in fewer reproductive health-promoting behaviors. This pattern—higher self-perceived knowledge coexisting with lower objective knowledge and lower engagement—may reflect miscalibrated perceived knowledge, or overconfidence, rather than accurate self-assessment (32). However, self-perceived knowledge was measured with a brief two-item scale, and our data cannot establish whether the discrepancy reflects miscalibration or, alternatively, differences in how respondents interpreted the items. Such a sense of knowledge sufficiency may nonetheless act as a barrier to behavior change, consistent with evidence that knowledge alone does not necessarily translate into behavior change (4). Conversely, structured preconception or contraceptive counseling has been shown to increase both knowledge and preventive behaviors, even before pregnancy (33, 34), suggesting that professional guidance may support behavior change. Because primary sources of reproductive health information among young adults tend to be informal—the Internet, social media, peers, or family rather than healthcare providers (35, 36)—there is a pressing need for structured educational programs and professional guidance.

Participants with no desire for future children were more likely to be unaware of preconception care. This aligns with previous findings that pregnancy intention is positively associated with engagement in preconception care behaviors (1, 37). However, considering the high rates of unintended pregnancy even in developed countries, education on preconception care should be inclusive of all individuals of reproductive age, regardless of their current family planning intentions.

Although several studies suggest that a higher level of knowledge leads to greater engagement in preconception care (37), our multivariable analysis did not confirm a significant association. This indicates that educational interventions alone may be insufficient to influence behavior. Rather than implying that education is unimportant, this suggests that education should be combined with motivational, behavioral, and service-linkage strategies to support behavioral adoption. This suggests that behavioral and motivational readiness—not knowledge alone—is the key to behavioral adoption. Future strategies must account for psychosocial and motivational dimensions alongside knowledge dissemination.

Moreover, the observed gender disparity, with males predominantly classified in the unaware group, should be interpreted with caution given the small number of male participants. This pattern may reflect persistent sociocultural norms that position reproductive health as a female responsibility. Integrating men into preconception health promotion is therefore essential for achieving equitable behavioral change, and future studies focusing specifically on men are needed to confirm and explain this pattern.

Finally, the stage-specific differences observed in this study may provide a useful descriptive basis for developing and evaluating stage-informed approaches in future intervention research.

### 4.1 Limitations

This study has several limitations. First, given its cross-sectional design, this study could not assess causal relationships between the PAPM stages and the influencing factors. Second, although we recruited participants through online noticeboards and social networking services to reduce convenience-sampling bias, the sample was recruited online and was heavily skewed toward females (83.9%); it likely had higher educational exposure and internet access and may not represent non-university, rural, lower-income, or digitally excluded young adults. Findings for male participants, in particular, are based on a small subgroup and should be regarded as preliminary, and broad claims about “emerging adults” in general should be made with caution. Third, the PAPM stages were collapsed into three groups and classified by self-report, which may not capture the distinction between deciding to act and acting and is subject to misclassification. In particular, self-perceived knowledge—the variable most strongly associated with the unaware group—was measured with only two self-appraisal items; although internal consistency was acceptable (α = 0.73), the validity of such a brief measure is limited, and our strongest association therefore rests on a weakly measured variable. In addition, the sexual-behavior items had smaller denominators (raising possible non-response bias), and the meal-skipping dietary subscale had low internal consistency (α = 0.51), although this subscale was used only descriptively and did not enter the regression models. Fourth, because preconception care was assessed mainly among those already sexually active, the findings may reflect a tendency to view it as relevant only after sexual debut, and we did not measure exposure to formal sex education, provider counseling, reproductive health services, or cultural and religious influences. Fifth, data were collected during the COVID-19 pandemic, which may have affected participants' health status and health-related and reproductive behaviors.

## 5 Conclusions

This study aimed to refine the understanding of preconception care behaviors by applying the PAPM stage model, with a focus on the early stages—unaware (Stage 1) and pre-adoption (Stages 2 and 3). This classification enabled the identification of factors influencing emerging adults' engagement in preconception care. Our findings suggest that different stages were characterized by different patterns of associated factors: awareness-raising for the unaware group, who reported high self-perceived knowledge despite limited engagement, and motivational and behavioral support for the pre-adoption group to move from awareness toward action.

## Data Availability

The original contributions presented in the study are included in the article/Supplementary material, further inquiries can be directed to the corresponding author.
