# Supplementary material for: Factors associated with preconception care behaviors and behavioral stages among emerging adults: a cross-sectional study
Source: Front Public Health. 2026 Aug 13;14:1842698. doi: 10.3389/fpubh.2026.1842698 (PMC13518509; doi:10.3389/fpubh.2026.1842698)
Supplement: Supplementary file 1 [file Table_1.docx]

**Table S1. PAPM staging questions, response options, and classification rules**

| **Question** | **Response option** | | **PAPM stage → Group** |
| --- | --- | --- | --- |
| Q1. Have you heard of preconception care? | No | | Stage 1 (Unaware) → **Unaware** |
|  | Yes → go to Q2 | |  |
| Q2. Do you currently engage in behaviors to improve your health or reproductive health for preconception care? | Yes | | Stage 6 (Acting) → **Adoption** |
|  | No → go to Q3 | |  |
| Q3. Which best describes your thoughts on preconception care? | I have never thought about it | | Stage 2 (Unengaged) → **Pre-adoption** |
|  | I am undecided whether to do it | | Stage 3 (Undecided) → **Pre-adoption** |
|  | I have decided not to do it | | Stage 4 (Decided not to act)  → *Excluded (n=2)* |
|  | | I have decided to do it | Stage 5 (Decided to act) → **Adoption** |

*Note.* Preconception care was defined for participants as selecting appropriate contraception when pregnancy is not desired, eating healthy food, exercising regularly, and taking folic acid before pregnancy. Group classification: Unaware = Stage 1; Pre-adoption = Stages 2–3; Adoption = Stages 5–6. Stage 4 (n = 2) was excluded from analysis owing to the very small number of observations.
